# 2-Meth­oxy-3-[(3,4,5-trimethoxy­anilino)methyl­idene]-3,4-dihydro-2*H*-1-benzopyran-4-one

**DOI:** 10.1107/S1600536809054889

**Published:** 2009-12-24

**Authors:** Magdalena Małecka, Michał Ciołkowski, Elżbieta Budzisz

**Affiliations:** a Department of Crystallography and Crystal Chemistry, University of Łódź, Pomorska 149/153, PL-90236 Łódź, Poland; b Department of Cosmetic Raw Materials Chemistry, Faculty of Pharmacy, Medical University of Łódź, Muszyńskiego 1, PL-90151 Łódź, Poland

## Abstract

The title mol­ecule, C_20_H_21_NO_6_, adopts a keto–amine tautomeric form. An intra­molecular N—H⋯O hydrogen bond, classified as a resonanse-assisted hydrogen bond, influences the mol­ecular conformation; the two benzene rings form a dihedral angle of 24.6 (1)°. In the crystal structure, weak inter­molecular C—H⋯O hydrogen bonds link mol­ecules into chains propagating along [001].

## Related literature

For the biological propertries of similar structures, see: Khan *et al.* (2009 ▶). For related structures, see: Gilli *et al.* (1994 ▶); Bertolasi *et al.* (1998 ▶); Małecka & Budzisz (2006 ▶); Małecka (2007 ▶).
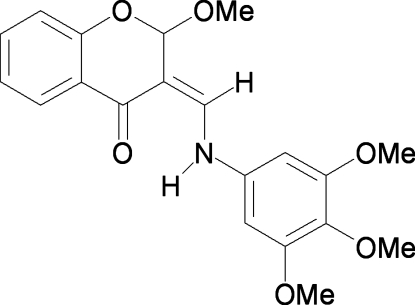


## Experimental

### 

#### Crystal data

C_20_H_21_NO_6_
*M* *_r_* = 371.38
Monoclinic, 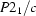

*a* = 11.6145 (6) Å
*b* = 20.8689 (9) Å
*c* = 7.3728 (5) Å
β = 94.533 (5)°
*V* = 1781.44 (17) Å^3^
*Z* = 4
Cu *K*α radiation
μ = 0.86 mm^−1^
*T* = 100 K
0.2 × 0.05 × 0.03 mm

#### Data collection

Oxford Diffraction Gemini E Ultra diffractometer
Absorption correction: multi-scan (*CrysAlis RED*; Oxford Diffraction, 2009 ▶) *T*_min_ = 0.844, *T*_max_ = 1.000
7511 measured reflections
2862 independent reflections
2344 reflections with *I* > 2σ(*I*)
*R*_int_ = 0.024
Standard reflections: 0

#### Refinement

*R*[*F* ^2^ > 2σ(*F* ^2^)] = 0.037
*wR*(*F* ^2^) = 0.101
*S* = 1.05
2862 reflections
248 parameters
H-atom parameters constrained
Δρ_max_ = 0.38 e Å^−3^
Δρ_min_ = −0.22 e Å^−3^

### 

Data collection: *CrysAlis CCD* (Oxford Diffraction, 2009 ▶); cell refinement: *CrysAlis RED* (Oxford Diffraction, 2009 ▶); data reduction: *CrysAlis RED*; program(s) used to solve structure: *SHELXS97* (Sheldrick, 2008 ▶); program(s) used to refine structure: *SHELXL97* (Sheldrick, 2008 ▶); molecular graphics: *PLATON* (Spek, 2009 ▶); software used to prepare material for publication: *PLATON*.

## Supplementary Material

Crystal structure: contains datablocks global, I. DOI: 10.1107/S1600536809054889/cv2678sup1.cif

Structure factors: contains datablocks I. DOI: 10.1107/S1600536809054889/cv2678Isup2.hkl

Additional supplementary materials:  crystallographic information; 3D view; checkCIF report

## Figures and Tables

**Table 1:** Hydrogen-bond geometry (Å, °)

*D*—H⋯*A*	*D*—H	H⋯*A*	*D*⋯*A*	*D*—H⋯*A*
N1—H1⋯O4	0.88	2.00	2.661 (2)	131
C14—H14*A*⋯O4^i^	0.98	2.48	3.414 (2)	160

Symmetry code: (i) 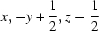.

**Fig. 1.**
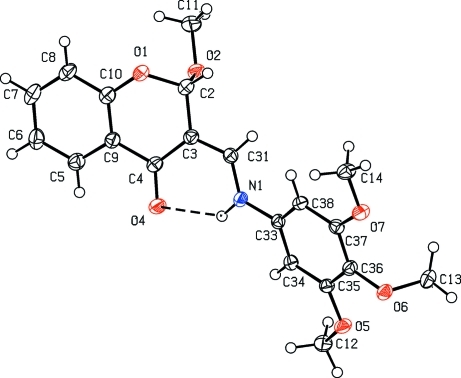
Molecular structure of the title compound, showing the atom-numbering scheme. Displacement ellipsoids are drawn at the 50% probability level. The N1—H1···O4 hydrogen bond classified as resonance assisted hydrogen bond (RAHB) is shown as dashed line.

## supplementary crystallographic information

### Comment

The present paper is a continuation of X-ray studies of a group of chromone derivatives (Małecka & Budzisz, 2006, Małecka, 2007). Due to their biological activity (Khan *et al.*, 2009) and the presence of intramolecular N—H···O hydrogen bond which could be classified as resonanse assisted hydrogen bond (RAHB) (Gilli *et al.*, 1994).

The more detailed insight into the molecular structure of the title compound shows that N—H···O hydrogen bond (Table 1) may be classified as resonance assisted hydrogen bond (RAHB). Such interactions RAHB have been investigated for homonuclear O—H···O interactions and for heteronuclear N—H···O RAHBs (Bertolasi *et al.*, 1998).

Considering O4═C4—C3═C31—N1—H1 fragment it is not observed the equalization of C4—C3 and C3═C31 in comparison to previuos examined structures However, it was found the elongation and shortening of C4═O4 and C3—N1 bonds, respectively. The π- electron delocalization effect is not so evident as in earlier investigated structures. It is associated with lack of the planarity and non-aromatic character of main part of molecule.

The packing of the molecules in the crystal lattice is stabilized *via* C—H···O hydrogen bonds (Table 1). Atom C14 is involved in a weak C—H···O intermolecular interaction with atom O4 related by symmetry *x*, 1/2 - *y*, -1/2 + *z* what results in forming chains C(11) along *c* axis.

The six-memebered pyrone ring adopts HC conformation, what confirm the Cremer & Pople parameters Q_T_= 0.396 (1) Å, φ_2_= 62.3 (2)°, θ_2_= 39.8 (3)°.

Bonds distances and angles are in a good agreement with expected values.

### Experimental

4-Oxo-4*H*-1-benzopyran-3-carboxaldehyde (0.348 g, 0.002 mol) was dissolved in hot toluene (20 ml) together with a small amount of *p*-toluenesulphonic acid as a catalyst. Resulting solution was kept under reflux while the solution of 3,4,5-trimethoxyaniline (0.366 g, 0.002 mol) in toluene (20 ml) was slowly added. When the addition was finished, the solution was kept under reflux for following 3 h. Then it was left in room temperature for 24 h. Approximately half of the solvent was removed under reduced pressure and resulting solution was left in the refrigerator for 48 h. Obtained precipitate was filtered off, added to 30 ml of methanol and refluxed for 30 minutes. The hot solution was filtered off to remove insolubilities. Then it was left in room temperature for 2 h. Approximately half of the solvent was removed under reduced pressure. Next the solution was left in the refrigerator for 4 days. Resulting precipitate was filtered off, washed with small amount of methanol and diethyl ether and dried under reduced pressure.

### Refinement

All H-atoms were positioned geometrically and refined with a riding model; for methyl H atoms *U*_iso_ were constrained to be 1.5 times *U*_eq_ of the carrier atom and C—H=0.98 Å; for others H atoms *U*_iso_ were constrained to be 1.2 times *U*_eq_ of the carrier atom and C—H=0.95 Å, 0.88 Å, for aromatic, amine groups, respectively. The incomplete data sets was collected due to poor quality, weakly diffracted crystal.

### Crystal data

**Table:**

C_20_H_21_NO_6_	*F*(000) = 784
*M**_r_* = 371.38	*D*_x_ = 1.385 Mg m^−^^3^
Monoclinic, *P*2_1_/*c*	Melting point: 408.2 K
Hall symbol: -P 2ybc	Cu *K*α radiation, λ = 1.54184 Å
*a* = 11.6145 (6) Å	Cell parameters from 4015 reflections
*b* = 20.8689 (9) Å	θ = 3.8–62.6°
*c* = 7.3728 (5) Å	µ = 0.86 mm^−^^1^
β = 94.533 (5)°	*T* = 100 K
*V* = 1781.44 (17) Å^3^	Needle, light yellow
*Z* = 4	0.2 × 0.05 × 0.03 mm

### Data collection

**Table:**

Oxford Diffraction Gemini E Ultra diffractometer	2862 independent reflections
Radiation source: fine-focus sealed tube	2344 reflections with *I* > 2σ(*I*)
graphite	*R*_int_ = 0.024
ω scans	θ_max_ = 62.7°, θ_min_ = 3.8°
Absorption correction: multi-scan (*CrysAlis RED*; Oxford Diffraction, 2009)	*h* = −13→12
*T*_min_ = 0.844, *T*_max_ = 1.000	*k* = −23→24
7511 measured reflections	*l* = −8→8

### Refinement

**Table:**

Refinement on *F*^2^	Primary atom site location: structure-invariant direct methods
Least-squares matrix: full	Secondary atom site location: difference Fourier map
*R*[*F*^2^ > 2σ(*F*^2^)] = 0.037	Hydrogen site location: inferred from neighbouring sites
*wR*(*F*^2^) = 0.101	H-atom parameters constrained
*S* = 1.05	*w* = 1/[σ^2^(*F*_o_^2^) + (0.0639*P*)^2^ + 0.2369*P*] where *P* = (*F*_o_^2^ + 2*F*_c_^2^)/3
2862 reflections	(Δ/σ)_max_ = 0.001
248 parameters	Δρ_max_ = 0.38 e Å^−^^3^
0 restraints	Δρ_min_ = −0.22 e Å^−^^3^

### Special details

**Table:**

Geometry. All e.s.d.'s (except the e.s.d. in the dihedral angle between two l.s. planes) are estimated using the full covariance matrix. The cell e.s.d.'s are taken into account individually in the estimation of e.s.d.'s in distances, angles and torsion angles; correlations between e.s.d.'s in cell parameters are only used when they are defined by crystal symmetry. An approximate (isotropic) treatment of cell e.s.d.'s is used for estimating e.s.d.'s involving l.s. planes.
Refinement. Refinement of *F*^2^ against ALL reflections. The weighted *R*-factor *wR* and goodness of fit *S* are based on *F*^2^, conventional *R*-factors *R* are based on *F*, with *F* set to zero for negative *F*^2^. The threshold expression of *F*^2^ > σ(*F*^2^) is used only for calculating *R*-factors(gt) *etc*. and is not relevant to the choice of reflections for refinement. *R*-factors based on *F*^2^ are statistically about twice as large as those based on *F*, and *R*- factors based on ALL data will be even larger.

### Fractional atomic coordinates and isotropic or equivalent isotropic displacement parameters (Å^2^)

**Table:**

	*x*	*y*	*z*	*U*_iso_*/*U*_eq_	
O1	1.23908 (9)	0.33541 (5)	0.31295 (15)	0.0255 (3)	
O4	0.95775 (10)	0.38347 (5)	0.58323 (17)	0.0289 (3)	
O2	1.09596 (10)	0.35060 (5)	0.07967 (15)	0.0260 (3)	
O5	0.47817 (9)	0.21086 (5)	0.59973 (16)	0.0249 (3)	
O6	0.53215 (9)	0.08807 (5)	0.56606 (15)	0.0238 (3)	
O7	0.74024 (10)	0.05173 (5)	0.46838 (16)	0.0244 (3)	
C2	1.12917 (14)	0.31326 (8)	0.2334 (2)	0.0235 (4)	
H2	1.1368	0.2676	0.1952	0.028*	
C3	1.03717 (14)	0.31789 (7)	0.3633 (2)	0.0210 (3)	
C4	1.03657 (14)	0.37260 (8)	0.4811 (2)	0.0222 (4)	
C5	1.14364 (15)	0.47322 (8)	0.5741 (2)	0.0275 (4)	
H5	1.0785	0.4873	0.6335	0.033*	
C6	1.24169 (16)	0.51097 (8)	0.5790 (2)	0.0310 (4)	
H6	1.2445	0.5507	0.6421	0.037*	
C7	1.33615 (15)	0.49006 (8)	0.4906 (2)	0.0316 (4)	
H7	1.4031	0.5163	0.4924	0.038*	
C8	1.33492 (15)	0.43197 (8)	0.3999 (2)	0.0285 (4)	
H8	1.4003	0.4181	0.3407	0.034*	
C9	1.13951 (14)	0.41457 (8)	0.4826 (2)	0.0226 (4)	
C10	1.23612 (14)	0.39430 (8)	0.3972 (2)	0.0239 (4)	
C31	0.95368 (14)	0.27129 (7)	0.3632 (2)	0.0205 (3)	
H31	0.9631	0.2339	0.2922	0.025*	
N1	0.86037 (11)	0.27528 (6)	0.45664 (18)	0.0204 (3)	
H1	0.8488	0.3121	0.5103	0.025*	
C33	0.77785 (14)	0.22652 (7)	0.4788 (2)	0.0189 (3)	
C34	0.66962 (14)	0.24532 (7)	0.5266 (2)	0.0201 (3)	
H34	0.6521	0.2894	0.5404	0.024*	
C35	0.58738 (13)	0.19877 (7)	0.5540 (2)	0.0197 (3)	
C36	0.61350 (14)	0.13378 (7)	0.5328 (2)	0.0209 (3)	
C37	0.72250 (14)	0.11622 (7)	0.4839 (2)	0.0202 (3)	
C38	0.80637 (14)	0.16237 (7)	0.4574 (2)	0.0198 (3)	
H38	0.8810	0.1503	0.4257	0.024*	
C12	0.44405 (15)	0.27643 (8)	0.6067 (2)	0.0270 (4)	
H12A	0.4956	0.2992	0.6964	0.041*	
H12B	0.3645	0.2791	0.6417	0.041*	
H12C	0.4486	0.2960	0.4867	0.041*	
C13	0.46540 (14)	0.06810 (8)	0.4033 (2)	0.0268 (4)	
H13A	0.4261	0.1053	0.3458	0.040*	
H13B	0.4079	0.0364	0.4343	0.040*	
H13C	0.5167	0.0490	0.3188	0.040*	
C14	0.85363 (14)	0.03145 (8)	0.4304 (2)	0.0270 (4)	
H14A	0.8719	0.0485	0.3122	0.040*	
H14B	0.8565	−0.0155	0.4275	0.040*	
H14C	0.9101	0.0474	0.5255	0.040*	
C11	1.18116 (17)	0.35023 (9)	−0.0509 (2)	0.0344 (4)	
H11A	1.2485	0.3752	−0.0039	0.052*	
H11B	1.1484	0.3693	−0.1651	0.052*	
H11C	1.2047	0.3060	−0.0731	0.052*	

### Atomic displacement parameters (Å^2^)

**Table:**

	*U*^11^	*U*^22^	*U*^33^	*U*^12^	*U*^13^	*U*^23^
O1	0.0172 (6)	0.0308 (6)	0.0284 (6)	−0.0008 (5)	0.0019 (5)	−0.0014 (5)
O4	0.0269 (7)	0.0251 (6)	0.0361 (7)	−0.0055 (5)	0.0123 (6)	−0.0058 (5)
O2	0.0259 (6)	0.0284 (6)	0.0240 (6)	−0.0016 (5)	0.0044 (5)	0.0024 (5)
O5	0.0189 (6)	0.0225 (6)	0.0343 (7)	0.0013 (4)	0.0078 (5)	−0.0024 (5)
O6	0.0221 (6)	0.0213 (6)	0.0285 (6)	−0.0054 (4)	0.0056 (5)	0.0009 (5)
O7	0.0229 (6)	0.0163 (5)	0.0349 (7)	0.0006 (4)	0.0080 (5)	0.0002 (5)
C2	0.0197 (8)	0.0256 (8)	0.0253 (9)	−0.0017 (6)	0.0021 (7)	0.0022 (7)
C3	0.0188 (8)	0.0237 (8)	0.0204 (8)	−0.0001 (6)	0.0013 (6)	0.0012 (6)
C4	0.0198 (8)	0.0234 (8)	0.0234 (8)	−0.0014 (6)	0.0028 (7)	0.0037 (7)
C5	0.0277 (10)	0.0290 (9)	0.0260 (9)	−0.0047 (7)	0.0037 (7)	0.0025 (7)
C6	0.0344 (10)	0.0290 (9)	0.0288 (9)	−0.0099 (7)	−0.0031 (8)	0.0020 (7)
C7	0.0222 (9)	0.0375 (10)	0.0343 (10)	−0.0095 (7)	−0.0037 (8)	0.0096 (8)
C8	0.0179 (9)	0.0379 (10)	0.0294 (9)	−0.0026 (7)	0.0003 (7)	0.0062 (8)
C9	0.0214 (9)	0.0266 (8)	0.0196 (8)	−0.0038 (6)	0.0003 (6)	0.0032 (6)
C10	0.0215 (9)	0.0286 (8)	0.0210 (8)	−0.0010 (7)	−0.0016 (7)	0.0059 (7)
C31	0.0209 (8)	0.0227 (8)	0.0178 (7)	−0.0009 (6)	0.0004 (6)	0.0004 (6)
N1	0.0188 (7)	0.0194 (6)	0.0233 (7)	−0.0031 (5)	0.0023 (6)	−0.0007 (5)
C33	0.0189 (8)	0.0215 (8)	0.0162 (7)	−0.0031 (6)	0.0001 (6)	0.0009 (6)
C34	0.0212 (9)	0.0187 (7)	0.0204 (8)	−0.0008 (6)	0.0009 (6)	−0.0008 (6)
C35	0.0174 (8)	0.0228 (8)	0.0189 (8)	0.0012 (6)	0.0024 (6)	−0.0007 (6)
C36	0.0199 (8)	0.0218 (8)	0.0209 (8)	−0.0033 (6)	0.0025 (6)	0.0014 (6)
C37	0.0223 (8)	0.0186 (7)	0.0198 (8)	0.0005 (6)	0.0025 (6)	−0.0001 (6)
C38	0.0172 (8)	0.0223 (8)	0.0201 (8)	0.0004 (6)	0.0033 (6)	0.0008 (6)
C12	0.0229 (9)	0.0250 (8)	0.0333 (9)	0.0057 (7)	0.0036 (7)	−0.0035 (7)
C13	0.0213 (9)	0.0224 (8)	0.0365 (10)	−0.0035 (6)	0.0009 (7)	0.0008 (7)
C14	0.0238 (9)	0.0216 (8)	0.0364 (10)	0.0042 (6)	0.0085 (8)	0.0000 (7)
C11	0.0363 (11)	0.0399 (10)	0.0284 (9)	−0.0068 (8)	0.0118 (8)	−0.0006 (8)

### Geometric parameters (Å, °)

**Table:**

O1—C10	1.379 (2)	C9—C10	1.395 (2)
O1—C2	1.438 (2)	C31—N1	1.332 (2)
O4—C4	1.251 (2)	C31—H31	0.9500
O2—C2	1.4042 (19)	N1—C33	1.416 (2)
O2—C11	1.434 (2)	N1—H1	0.8800
O5—C35	1.3614 (19)	C33—C34	1.389 (2)
O5—C12	1.4267 (19)	C33—C38	1.391 (2)
O6—C36	1.3781 (19)	C34—C35	1.388 (2)
O6—C13	1.438 (2)	C34—H34	0.9500
O7—C37	1.3677 (18)	C35—C36	1.401 (2)
O7—C14	1.4318 (19)	C36—C37	1.393 (2)
C2—C3	1.493 (2)	C37—C38	1.394 (2)
C2—H2	1.0000	C38—H38	0.9500
C3—C31	1.373 (2)	C12—H12A	0.9800
C3—C4	1.435 (2)	C12—H12B	0.9800
C4—C9	1.481 (2)	C12—H12C	0.9800
C5—C6	1.383 (2)	C13—H13A	0.9800
C5—C9	1.397 (2)	C13—H13B	0.9800
C5—H5	0.9500	C13—H13C	0.9800
C6—C7	1.390 (3)	C14—H14A	0.9800
C6—H6	0.9500	C14—H14B	0.9800
C7—C8	1.384 (3)	C14—H14C	0.9800
C7—H7	0.9500	C11—H11A	0.9800
C8—C10	1.390 (2)	C11—H11B	0.9800
C8—H8	0.9500	C11—H11C	0.9800
			
C10—O1—C2	114.66 (12)	C34—C33—N1	117.44 (13)
C2—O2—C11	112.27 (13)	C38—C33—N1	120.61 (14)
C35—O5—C12	116.97 (12)	C35—C34—C33	119.10 (14)
C36—O6—C13	112.60 (12)	C35—C34—H34	120.4
C37—O7—C14	117.01 (12)	C33—C34—H34	120.4
O2—C2—O1	109.21 (12)	O5—C35—C34	124.86 (14)
O2—C2—C3	108.48 (13)	O5—C35—C36	114.91 (13)
O1—C2—C3	112.02 (13)	C34—C35—C36	120.23 (15)
O2—C2—H2	109.0	O6—C36—C37	120.92 (14)
O1—C2—H2	109.0	O6—C36—C35	119.52 (14)
C3—C2—H2	109.0	C37—C36—C35	119.53 (14)
C31—C3—C4	121.76 (15)	O7—C37—C36	115.26 (14)
C31—C3—C2	119.67 (14)	O7—C37—C38	123.80 (14)
C4—C3—C2	118.53 (14)	C36—C37—C38	120.93 (14)
O4—C4—C3	123.18 (14)	C33—C38—C37	118.27 (15)
O4—C4—C9	121.12 (15)	C33—C38—H38	120.9
C3—C4—C9	115.62 (14)	C37—C38—H38	120.9
C6—C5—C9	120.58 (17)	O5—C12—H12A	109.5
C6—C5—H5	119.7	O5—C12—H12B	109.5
C9—C5—H5	119.7	H12A—C12—H12B	109.5
C5—C6—C7	119.20 (17)	O5—C12—H12C	109.5
C5—C6—H6	120.4	H12A—C12—H12C	109.5
C7—C6—H6	120.4	H12B—C12—H12C	109.5
C8—C7—C6	121.54 (16)	O6—C13—H13A	109.5
C8—C7—H7	119.2	O6—C13—H13B	109.5
C6—C7—H7	119.2	H13A—C13—H13B	109.5
C7—C8—C10	118.64 (16)	O6—C13—H13C	109.5
C7—C8—H8	120.7	H13A—C13—H13C	109.5
C10—C8—H8	120.7	H13B—C13—H13C	109.5
C10—C9—C5	119.05 (15)	O7—C14—H14A	109.5
C10—C9—C4	119.66 (15)	O7—C14—H14B	109.5
C5—C9—C4	121.24 (15)	H14A—C14—H14B	109.5
O1—C10—C8	117.42 (15)	O7—C14—H14C	109.5
O1—C10—C9	121.56 (14)	H14A—C14—H14C	109.5
C8—C10—C9	120.98 (16)	H14B—C14—H14C	109.5
N1—C31—C3	123.98 (15)	O2—C11—H11A	109.5
N1—C31—H31	118.0	O2—C11—H11B	109.5
C3—C31—H31	118.0	H11A—C11—H11B	109.5
C31—N1—C33	126.91 (13)	O2—C11—H11C	109.5
C31—N1—H1	116.5	H11A—C11—H11C	109.5
C33—N1—H1	116.5	H11B—C11—H11C	109.5
C34—C33—C38	121.93 (14)		

### Hydrogen-bond geometry (Å, °)

**Table:**

*D*—H···*A*	*D*—H	H···*A*	*D*···*A*	*D*—H···*A*
N1—H1···O4	0.88	2.00	2.661 (2)	131
C14—H14A···O4^i^	0.98	2.48	3.414 (2)	160

Symmetry codes: (i) *x*, −*y*+1/2, *z*−1/2.
